# Milk-derived extracellular vesicles and gut health

**DOI:** 10.1038/s41538-025-00375-1

**Published:** 2025-01-30

**Authors:** Barathan Muttiah, Jia Xian Law

**Affiliations:** Department of Tissue Engineering and Regenerative Medicine, Faculty of Medicine, Universiti Kebangsaan Malaysia, Cheras, Kuala Lumpur Malaysia

**Keywords:** Biotechnology, Biomarkers

## Abstract

Milk is a nutrient-rich liquid produced by mammals, offering various health benefits due to its composition of proteins, fats, carbohydrates, vitamins, and minerals. Beyond traditional nutritional aspects, recent research has focused on extracellular vesicles (EVs) found in milk and their potential health benefits, especially for gastrointestinal (GI) health. Milk-derived EVs have been shown to influence gut microbiota, promote gut barrier integrity, support tissue repair and regeneration, modulate immune responses, and potentially aid in managing conditions like inflammatory bowel disease (IBD) and colorectal cancer. This review discusses the current understanding of milk-EVs’ effects on gut health, highlighting their potential therapeutic applications and future research directions. These findings underscore the promising role of milk-derived EVs in advancing GI health and therapeutics, paving the way for innovative approaches in oral drug delivery and targeted treatments for GI disorders.

## Introduction

Milk is a nutrient-rich liquid produced by mammals, primarily to nourish their offspring. It serves as a complete and natural food source, containing a balanced combination of proteins, fats, carbohydrates, vitamins, and minerals^1^. While milk is commonly associated with dairy farming and cow’s milk, it is important to note that various mammals, including goats, sheep, buffalo, and even humans, produce milk that is consumed by their respective species. Milk can be processed into various forms, such as whole milk, skim milk, and various dairy products like yogurt, cheese, and butter. The processing methods can alter the nutritional content and taste of the final products.

Milk is widely consumed worldwide as it provides a variety of health benefits^2,3^. Primarily, it stands out as an abundant source of calcium, imperative for fostering the development and resilience of robust bones and teeth. Furthermore, its composition encompasses high-quality proteins replete with essential amino acids, pivotal for the promotion of muscle growth, repair, and holistic bodily upkeep. Additionally, milk serves as a commendable reservoir of various vitamins and minerals, playing indispensable roles in bolstering immune function and sustaining overall health. Beyond these primary benefits, milk boasts numerous other nutritional values, making it a versatile and wholesome dietary choice.

The gut, which is the gastrointestinal (GI) system that includes the stomach, intestines, and colon is the first organ system that expose to the consumed milk. Thus, it is probably that first organ system that will benefit from milk consumption. Generally, milk is rich in nutrients that are vital for the proper functioning of the GI system. Studies have found that milk lactose can modify the gut microbiome by increasing the beneficial taxa such as *Bifidobacterium* and *Lactobacillus* and increasing the secretion of healthy metabolites such as acetate and lactate^4,5^. A healthy gut microbiome is important in supporting nutrient absorption and immune modulation^6,7^. Certain milk components, such as the immunoglobulins, polyunsaturated fatty acid, and lactoferrin, have been reported to reduce gut inflammation^8,9^. Milk is also rich in calcium and vitamin D that facilitate calcium absorption. Higher calcium absorption has been linked with lower risk of colorectal cancer^10^. Furthermore, whey protein in milk is found to improve the gut barrier function and the high water content in milk assists in digestion and prevents constipation^11^. Previous study also found that milk proteins can improve the gastric emptying and GI transit^12^.

While milk is a valuable source of nutrients, it is important to note that some individuals may be lactose intolerant or have allergies to milk proteins. Thus, research has been conducted to identify the components of milk which provide the health benefits, so that those with lactose intolerant or allergic to milk proteins also can garner the benefits. One of the milk components that has received a lot of attention nowadays is the extracellular vesicles (EVs). Milk-EVs from many different animal sources, including human^13^, cow^14^, goat^15^, horse^16^, pig^17^, and camel^18^, have been investigated in previous studies.

EVs are small, membrane-enclosed particles released by cells into the extracellular space^19^. These vesicles play a crucial role in intercellular communication, facilitating the transfer of bioactive molecules between cells^20^. EVs are involved in a wide range of physiological processes and are implicated in various pathological conditions. There are three main types of EVs, i.e., exosomes, microvesicles and apoptotic bodies, that differ in size, biogenesis, release pathway, cargo, and function^21^. The overlap in size and lack of specific biomarkers render the isolation of specific type of EVs a challenging task. In fact, most of the isolation methods such as tangential flow filtration, size-exclusion chromatography, differential centrifugation, and ultrafiltration separate the EVs based on its size^22,23^. EVs can be found in many body fluids including milk^24^. They are rich in proteins, lipids and nucleic acids (such as RNA and DNA)^25^. The contents of these vesicles reflect the originating cell’s status and can have functional implications when transferred to recipient cells. This transfer of bioactive molecules allows EVs to influence various physiological processes, such as immune response modulation, tissue repair, and the regulation of cellular functions^26^.

Milk-EVs have been observed to function similarly to milk, offering comparable benefits. Studies showed that milk-EVs can stimulate the maturation of immune cells, modulate the immune response, enhance neuronal synapses formation, improve the health of GI system, and play a role in metabolic regulation^27–29^. The importance of milk-EVs is highlighted by the fact that breast milk provides more beneficial effects to the infant compared to infant milk formulation made from cows’ milk that was found to be scarce in EVs^30,31^.

**Fig. 1:**
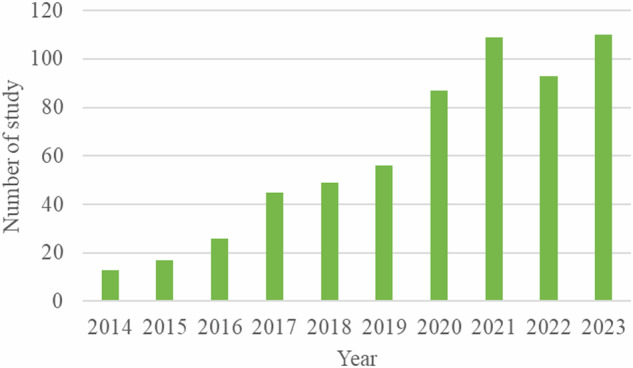
Trends in milk-EVs research. The figure illustrates the number of studies found in PubMed when searching with the keywords “milk” and “extracellular vesicles” as of December 20, 2023. The figure highlights the number of publications over time, reflecting the trajectory of research in this niche. The growing body of research captured in the figure underscores the importance of milk as a source of bioactive EVs and supports further exploration into their roles in health and disease. Analyzing the trend in publication numbers can help identify periods of heightened research activity, emerging interests, and potential gaps in the literature. This information is particularly useful for researchers and industry stakeholders interested in the nutritional and therapeutic implications of milk-derived EVs.

Nowadays, there is a growing interest in milk-EVs, reflected by the increasing number of publications related to milk-EVs in the past 10 years. Figure 1 shows the number of publications related to milk-EVs over the past 10 years. This review will explore the effects of milk-EVs on gut health. Maintenance of healthy gut is very important as mutual interaction between gut and other organ systems such as brain^32^, liver^33^, and immune system^34^ has huge influence on the host health and disease. In addition, the content milk-EVs and potential of milk-EVs as drug carriers are also briefly discussed. Milk-EVs have great potential as vehicles for oral delivery of drugs, especially those that are susceptible to enzymatic digestion, as it has low immunogenicity, excellent biocompatibility, resist enzymatic degradation and acidic environment to protect the cargo, and efficiency absorption in the GI system^35,36^.

## Content of milk-derived extracellular vesicles

Milk contains a high number of EVs, with a plethora of biological molecules located both within the vesicles (cytosolic) and on their membrane (transmembrane or bound to the lipid-bilayer membrane). Much like EVs found in other bodily fluids, milk-EVs are abundant in proteins, lipids, and nucleic acids, encompassing both RNA and DNA^27^. To date, the DNA content of milk-EVs has not been reported in existing studies. The quantity and content of EVs undergo changes during the processing of milk into various forms. For instance, Turner et al. discovered that infant milk formulation made from cows’ milk contains a lower concentration of EVs compared to raw cow milk^31^. Furthermore, in the same investigation, it was revealed that although 90% of milk-EV miRNAs are retained in infant milk formulation-EVs, only 20% of milk-EV proteins are identified in the corresponding infant milk formulation-EVs. These findings suggest that while miRNAs are preserved during the production of infant milk formulation, proteins exhibit a lower retention rate. In addition, homogenization and thermal processing (low-temperature heat, pasteurization and ultra-high-temperature) of cow milk led to over 60% reduction in EV concentration^37^.

### Proteins

Turner et al. conducted a comprehensive analysis, identifying 365, 1084, and 343 proteins in human milk-EVs, cow milk-EVs, and infant milk formulation-EVs, respectively^31^. The examination revealed that 216 proteins were shared between human milk-EVs and cow milk-EVs, while 113 proteins were notably more abundant in cow milk-EVs, and 17 proteins were more prevalent in human milk-EVs. In a separate study, the authors identified 229 and 239 proteins in human and cow milk-EVs, respectively, with only 53 proteins common to both types of milk-EVs^38^. Notably, other studies reported a considerably higher range of proteins, ranging from 1974 to 9430, in cow milk-EVs^39–42^. Similarly, Herwijnen et al. identified 1963 proteins in human milk-EVs^43^. Further investigations by Samuel et al. highlighted variations in protein composition among different stages of lactation, with 24-h, 48-h, 72-h colostrum, and mature milk-EVs displaying 1264, 1404, 963, and 1306 unique proteins, respectively^40^. Fluctuations in protein content of milk-EVs collected at different lactation stages also have been reported in other studies^44,45^. Furthermore, the animal health status will also affect the protein cargo of milk-EVs. For instance, a study revealed 1516 proteins in healthy cow milk-EVs, while only 1330 proteins were identified in milk-EVs from cows infected with bovine leukemia virus^46^. Among these, 304 proteins were unique to healthy cow milk-EVs, and 118 were specific to infected cow milk-EVs. Additionally, protein analyses of milk-EVs from pig and horse sources have been reported in various studies^16,47,48^, as outlined in Table 1.

**Table 1:** List of studies examined the protein profile of milk-EVs isolated from different animal species

Reference	Species	EV isolation method	Analytic method	Number of protein identified
Turner et al.^31^	Human and cow	UC and SEC	LC-MS/MS	Human: 365 Cow: 1084 IMF: 343
Vaswani et al.^38^	Human and cow	SEC	LC-MS/MS	Human: 229 Cow: 239
Yang et al.^45^	Human and cow	UC, filtration, and sucrose gradient centrifugation	LC-MS/MS	920
Herwijnen et al.^43^	Human	Density gradient UC	LC-MS/MS	1963
Reinhardt et al.^39^	Cow	Density gradient UC	LC-MS/MS	2107
Rahman et al.^46^	Cow	Filtration and UC	LC-MS/MS	1516
Rahman et al.^42^	Cow	Filtration and UC	nanoLC-MS/MS	2225
Grossen et al.^47^	Cow	Sequential UC and density gradient centrifugation	LC-MS/MS	817
Brown et al.^129^	Cow	UC	LC-MS/MS	162
Samuel et al.^40^	Cow	UC and density gradient UC	LC-MS/MS	9340
Benmoussa et al.^41^	Cow	Differential UC	LC-MS/MS	1974
Chen et al.^48^	Pig	UC	LC-MS/MS	639
Ferreira et al.^44^	Pig	UC and SEC	LC-MS/MS	319
Sedykh et al.^16^	Horse	UC	LC-MS/MS	NA

*UC* ultracentrifugation, *SEC* size-exclusion chromatography, *LC-MS/MS* liquid chromatography with tandem mass spectrometry, *IMF* infant milk formulation, *NA* not available.

Notably, several proteins in milk-EVs have been identified for their immunomodulatory functions. For example, lactoferrin, a well-known protein in milk-EVs, has antimicrobial and anti-inflammatory properties that contribute to the immune protection provided by milk^49^. In addition, Giovanazzi et al. found higher CD81 signals in milk-EVs compared to those in other biofluids, such as serum. The increased presence of CD81 might influence the stability or cargo-carrying capacity of milk-EVs, as CD81 is involved in cell adhesion and signal transduction^50^. Cytosolic proteins, such as annexins and Ras-related proteins, were identified in all milk-EV samples. Furthermore, the high enrichment of Rab proteins and annexins in bovine milk exosomes underscores their critical roles in orchestrating vesicle fusion, stability, and trafficking events, which are essential for the efficient delivery of bioactive molecules to recipient cells^51^. Animal studies show bovine milk-EVs contain TGF-β, crucial for mucosal barrier formation and Th17 phenotype promotion^52^. Heat shock cognate 70, found in bovine milk exosomes, plays a role in maintaining protein homeostasis under both stressed and non-stressed conditions^53^. Pig milk-EVs also carry proteins like growth factors, enzymes, extracellular matrix proteins, and immune-related proteins linked to immune function, acute inflammatory response, and B cell-mediated immunity^48^. Understanding these functional aspects aids in leveraging the benefits of EVs and their potential applications based on their protein content and sources, amidst variables such as species, diet, health status, and milk processing methods^27^. Table 2 summarizes the identified proteins in milk-EVs and their functions.

**Table 2:** Important proteins in milk-EVs and their functions

Protein	Source	Functions	Reference
Lactoferrin	Milk-EVs	Antimicrobial, anti-inflammatory	^49^
CD81	Milk-EVs	Cell adhesion, signal transduction, potentially influence stability and cargo capacity	^50^
Annexins	Milk-EVs	Vesicle fusion, stability, trafficking	^51^
Ras-related proteins	Milk-EVs	Various intracellular signaling pathways	^51^
Rab proteins	Bovine milk-EVs	Vesicle fusion, stability, trafficking	^51^
TGF-β	Bovine milk-EVs	Mucosal barrier formation, Th17 phenotype promotion	^52^
Heat shock cognate 70	Bovine milk-EVs	Maintains protein homeostasis under stressed and non-stressed conditions	^53^
Various growth factors	Pig milk-EVs	Cell growth and differentiation	^48^
Enzymes	Pig milk-EVs	Catalyze biochemical reactions	^48^
Extracellular matrix proteins	Pig milk-EVs	Structural support and signaling in tissue architecture	^48^
Immune-related proteins	Pig milk-EVs	Immune function, acute inflammatory response, B cell-mediated immunity	^48^

### RNAs

A comparative study examining miRNAs in human milk-EVs, cow milk-EVs, and infant milk formulation-EVs detected 832, 1357, and 1513 miRNAs, respectively^31^. A noteworthy finding was the presence of 739 common miRNAs across all samples. In a distinct investigation, 349, 341, and 379 miRNAs were identified in human, cow, and goat milk-EVs, respectively^54^. Similar to the previous study, the milk-EVs from diverse animal sources exhibited highly similar miRNA profiles, with 323 miRNAs (81%) common to human and bovine milk-EVs and 318 miRNAs (74%) shared between human and goat milk-EVs. Moreover, the study found that the milk-EV miRNAs survive the pasteurization process whereby 87% and 94% of milk-EV miRNAs identified in both pasteurized and unpasteurized cow and goat milk-EVs, respectively. However, infant milk formulation-EVs were reported to have significantly fewer miRNAs compared to human milk-EVs. Similar findings were corroborated by Leiferman et al., who detected 221 miRNAs in human milk-EVs but none in infant milk formulation-EVs, revealing that exosome-sized vesicles isolated from the latter are casein micelles instead of exosomes^55^. Yun et al. reported a high conservation of the top 10 most abundantly expressed milk-EV miRNAs across different species, including humans, cows, and goats, tested in their study^56^. In addition, they found ~60–70% similarity in milk-EV miRNAs between colostrum and mature milks collected from humans, cows, and goats. Cai et al. identified 492 known miRNAs in cow milk-EVs, with 18 of them differentially expressed between milk-EVs collected from healthy cows and those infected with *Staphylococcus aureus*^57^. The miRNA content of milk-EVs is also influenced by stress. A study revealed that relocation stress significantly alters the expression levels of 13 out of 69 identified miRNAs in cow milk-EVs^53^. Despite the diversity in identified miRNAs across these studies, it is noteworthy that the top 10 most abundant miRNAs accounted for the majority (70–90%) of the total sequencing reads^55,57^.

Research on non-miRNA RNAs in milk-EVs is relatively less common compared to miRNAs. Notably, more than 13,000 and 19,000 mRNAs have been detected in pig milk-EVs and cow milk-EVs, respectively^48,58,59^. The exploration of long noncoding RNAs (lncRNAs) in cow milk-EVs has identified over 3000 lncRNAs^60,61^. Similarly, studies on pig milk-EVs have detected more than 3000 lncRNAs, along with 61 circular RNAs (circRNAs)^62^. In the case of cow milk-EVs, a substantial number of circRNAs, specifically 2059 and 22,107 circRNAs, were identified in two separate studies^59,61^. Intriguingly, these studies revealed that while mRNAs are consistently present in both colostrum and mature milk-EVs, the circRNA profiles significantly differ between these two milk types. In addition, various other RNA types have been found in milk-EVs, including transfer RNA (tRNA), ribosomal RNA (rRNA), small nuclear RNA (snRNA), small nucleolar RNA (snoRNA), small cytoplasmic RNA (scRNA), and signal recognition particle RNA (SRP RNA)^63–65^. The studies that have extensively profiled the RNA content of milk-EVs are outlined in Table 3.

**Table 3:** List of studies examined the RNA cargo of milk-EVs isolated from different animal species

Reference	Species	EV isolation method	Analytic method	Type and number of RNA identified
Turner et al.^31^	Human and cow	UC and SEC	NGS	miRNA Human: 832 Cow: 1357 IMF: 1513
Golan-Gerstl et al.^54^	Human, goat, and cow	ExoQuick	NGS	miRNA Human: 349 Cow: 341 Goat: 379
Yun et al.^56^	Human, goat, and cow	Filtration and UC	NGS	miRNA Human: 349 Cow: 341 Goat: 379
Leiferman et al.^55^	Human and cow	Differential UC	NGS	miRNA Human: 221 IMF: undetectable
Zhou et al.^82^	Human	ExoQuick	NGS	miRNA 602
Li et al.^130^	Cow	Umbio Exosome Isolation kit	NGS	miRNA 492
Sun et al.^63^	Cow	Sucrose gradient centrifugation and filtration	NGS	miRNA 417
Gu et al.^131^	Pig	ExoQuick	NGS	miRNA 180
Chen et al.^64^	Pig	UC	NGS	miRNA 176
Cai et al.^57^	Cow	UC	NGS	miRNA 492
Colitti et al.^53^	Cow	exoEasy	NGS	miRNA 69
Izumi et al.^58^	Cow	UC	miRNA and mRNA microarray	miRNA 79 mRNA 19,320
Benmoussa et al.^65^	Cow	UC	NGS	Small RNAs are mainly composed of miRNAs
Chen et al.^48^	Pig	UC	NGS	mRNA 13,895
Wang et al.^59^	Cow	ExoQuick	NGS	mRNA 18,612 circRNA 2059
Ma et al.^61^	Cow	UC	NGS	circRNA 22,107
Zeng et al.^62^	Pig	UC	NGS	lncRNA 3275 circRNA 61
Zeng et al.^60^	Cow	UC	NGS	lncRNA 3481

*UC* ultracentrifugation, *SEC* size-exclusion chromatography, *NGS* next-generation sequencing, *IMF* infant milk formulation, *RNAs* ribonucleic acid, *miRNA* microRNA, *mRNA* messenger RNA, *lncRNA* long noncoding RNA, *circRNA* circular RNA.

### Lipids

Grossen et al. studied the lipid composition of cow milk-EVs and identified more than 200 fatty acid variations belonging to eight major lipid classes^47^. Notably, high levels of cholesterol, phosphatidylcholine, phosphatidylserine, and phosphatidylethanolamine were detected in this study. In a study by Blans et al., the lipid profiles of both human and cow milk-EVs were examined, revealing a higher percentage of sphingomyelin and phosphatidylethanolamine compared to phosphatidylcholine and phosphatidylserine^66^. Yassin et al. reported that camel milk-EVs contain the highest amount of phosphatidylcholine, followed by phosphatidylethanolamine, phosphatidylserine, and phosphatidylinositol, resembling the lipidomic profile of cow milk-EVs^67^. Chen et al. detected 395 lipids in human milk-EVs, with phosphatidylethanolamine, phosphatidylcholine, and phosphatidylserine showing the highest abundance^68^. The presence of these phospholipids in milk-EVs is crucial as they play a significant role in brain development^69,70^. The exploration of lipid composition in milk-EVs provides valuable insights into their potential functional roles and nutritional significance. Table 4 shows lipid profile of milk-EVs isolated from different animal species.

**Table 4:** List of studies examined the lipid profile of milk-EVs isolated from different animal species

Reference	Species	EV isolation method	Analytic method	Lipid components
Grossen et al.^47^	Cow	Sequential UC and density gradient centrifugation	MS-based lipid analysis	Rich in cholesterol, phosphatidylcholine, phosphatidylserine, and phosphatidylethanolamine
Blans et al.^66^	Human and cow	SEC	Thin layer chromatography	Richer in sphingomyelin and phosphatidylethanolamine compared to phosphatidylcholine and phosphatidylserine
Yassin et al.^67^	Camel and cow	Differential UC	HPLC	Contains the highest amount of phosphatidylcholine, followed by phosphatidylethanolamine, phosphatidylserine, and phosphatidylinositol
Chen et al.^68^	Human	UC and filtration	LC-MS/MS	Rich in phosphatidylethanolamine, phosphatidylcholine and phosphatidylserine

*MS* mass spectrometry, *UC* ultracentrifugation, *HPLC high-performance* liquid chromatography, *LC-MS/MS* liquid chromatography with tandem mass spectrometry.

## Gastrointestinal system

At the core of the GI system often known as the digestive system lies the intestinal epithelium, a single layer of cells lining the intestine’s surface. This epithelium forms a vital barrier between the body’s internal environment and the contents of the gut, performing essential functions in digestion, nutrient uptake, and immune protection^71^. Structurally, the small intestine features villi and crypts within the intestinal epithelium, which increase surface area for enhanced absorption. The epithelial cells undergo continuous renewal through cellular turnover, where stem cells located at the bases of crypts differentiate into enterocytes, goblet cells, enteroendocrine cells, and Paneth cells. This tightly regulated process ensures the integrity of the intestinal barrier, crucial for blocking harmful substances while facilitating nutrient and water absorption. Its functions extend beyond nutrient absorption, encompassing crucial roles in immune defense and the maintenance of overall physiological well-being^72^. The presence of a substantial portion of the body’s immune cells in the gut underscores its significance in immune function^73^. In addition, the GI tract hosts a diverse community of microorganisms, collectively known as the gut microbiota, contributing to digestion, metabolism, and overall health^74^. Disruptions in the balance of intestinal epithelial cells can lead to conditions such as inflammatory bowel diseases (IBD), leaky gut syndrome, and malabsorption disorders, highlighting the importance of studying and understanding the processes that govern the health and function of the intestinal epithelium^75^.

Autophagy plays a crucial role in maintaining intestinal homeostasis by managing cell stress and facilitating tissue regeneration. The intestinal mucosa faces constant exposure to antigens and mechanical stress, requiring an intact barrier and healthy microbiota to prevent infection and inflammation^76^. This involves rapid cell turnover every 4–5 days, balanced proliferation and apoptosis of stem cells in the intestinal crypts, and the maintenance of tight junctions to regulate permeability. Goblet cells secrete mucus to protect the epithelium, and the epithelium interacts with gut-associated lymphoid tissue to maintain immune tolerance to commensal bacteria and defend against pathogens. In addition, the epithelium has a symbiotic relationship with the gut microbiota, aiding in digestion and immune function. Enterocytes efficiently absorb nutrients from the gut lumen, while antimicrobial peptides are secreted to control microbial populations^77,78^. Disruptions in this delicate balance can lead to GI disorders such as inflammatory bowel diseases (IBD), celiac disease, and increased susceptibility to infections^79^.

The journey of food through the GI system illustrates the importance of the intestinal epithelium. Food ingestion initiates mechanical and enzymatic breakdown during chewing and salivation, forming a mixture called bolus. The bolus progresses to the stomach via the esophagus, where gastric juices and enzymes convert it into a semi-liquid substance known as chyme. As chyme enters the small intestine, digestive enzymes and bile break down complex nutrients, facilitating their absorption through the intestinal lining. The absorbed nutrients—carbohydrates, proteins, fats, vitamins, and minerals—are transported through the bloodstream to various organs and tissues, supporting energy production and cellular functions. The remaining undigested material, along with water, travels to the large intestine, where water and electrolytes are absorbed, forming feces from the indigestible components^80,81^.

## Absorption of milk-derived extracellular vesicles in the gastrointestinal system

Research on the absorption of milk-EVs in the GI system is an ongoing area, with the understanding of this process continuously evolving. Moreover, further studies are required to clarify the significance of milk-EV absorption in the GI system. When it comes to absorption in the GI system, the fate of these vesicles depends on several factors. The foremost consideration is their stability in the GI environment, where EVs must endure the harsh conditions of the stomach, encompassing low pH and the presence of digestive enzymes. Milk-EVs have been reported to resist degradation in the stomach as they are resilient to low pH, high temperature, repeated freezing and thawing, and enzymatic degradation^60,82–86^. This resilience is likely attributed to the protective lipid-bilayer membrane surrounding the EV cargo. Notably, milk-EVs have been observed to exploit the pH gradient between the intestinal lumen and the circulation system, thereby enhancing transcytosis^87^.

Second, the vesicles must possess the capability to traverse the intestinal epithelium for absorption to take place. The exact mechanism behind this process remains incompletely understood but may involve endocytosis or alternative cellular uptake processes^88,89^. Milk-EVs have been reported to escape the endo-lysosomal confinement via fusion with endo/lysosome membrane^87^. Several studies indicate that intestinal epithelial cells take up milk-EVs, facilitating their subsequent transport to various tissues^90–92^. After oral administration, these EVs have been reported to be transported through the circulatory system, accumulating in organs such as the liver, spleen, kidney, heart, and lungs within six hours^93^. In the same investigation, it was observed that the neonatal Fc receptor (FcRn), which aids in the adsorption of Fc-targeted particles in the GI system, is involved in the adsorption of milk-EVs. In addition, galectin-3 expressed by intestinal cells was found to enhance the internalization of bovine milk-EVs^94^. The activation of the ERK1/2 and p38 MAPK pathway has been associated with a more efficient apical-to-basolateral transportation of milk-EVs, stimulating the translocation of GLUT-2 to the apical membrane of the intestinal epithelium^87^. Because of their resistance to enzymatic digestion and their ability to permeate the intestinal epithelium, milk-EVs have been explored as a vehicle for oral drug delivery^95^. For example, they have been utilized in the oral delivery of paclitaxel to tumors in an in vivo model of lung tumor xenograft^90^, as well as for the oral delivery of nucleic acid therapies in another study^96^.

Third, understanding the interaction between milk-EVs and the mucus and gut microbiota is crucial. The presence of mucus and a diverse microbiota in the GI tract can potentially impact the stability, bioavailability, and absorption of EVs. Notably, milk-EVs demonstrate efficient crossing of the intestinal mucosa^87^. This discovery holds significance, considering that the mucus layer is widely recognized as a barrier that impedes the absorption of most orally delivered nanoparticles^97^. Furthermore, milk-EVs have been identified as agents that can alter the composition of gut microbiota and modulate the integrity of the mucus barrier^98,99^. Mucus penetration ability of milk-EVs has been enhanced via coating with polyethylene glycol (PEG)^100^.

Fourth, it has been demonstrated that milk-EVs exhibit immunomodulatory effects in the gut, impacting the activity of immune cells and potentially influencing EV absorption. This idea is substantiated by evidence of the absorption of cow milk-EVs by intestinal dendritic cells and macrophages located in the lamina propria, leading to the restoration of gut barrier function^88^. Changes in gut barrier function can have implications for EV absorption, as nanoparticles are known to get trapped in the mucus layer, hindering their absorption on the surface of the intestinal epithelium^101^.

Lastly, it is worth noting that milk-EVs originating from different animal species may exhibit varying absorption and bioavailability. This distinction was highlighted in a study that revealed distinct tissue distribution patterns for orally ingested milk-EVs from allogeneic and xenogeneic animal species^102^. Specifically, human milk-EVs were reported to be more potent in stimulating skeletal muscle growth and performance compared to cow milk-EVs^103^.

## Benefits of milk-EVs to the gastrointestinal system

### Bioactive molecules delivery to maintain tissue homeostasis

Milk-EVs are rich in bioactive molecules such as proteins, lipids, and nucleic acid which could potentially play a role in supporting various cellular function and signaling pathways within the GI system^51^. For example, milk-EVs have been found to promote the intestinal epithelial cell growth^104–106^, support intestinal tract development by increasing the villus height and crypt depth^105^, and reduce intestinal epithelial cell death by protect them against oxidative stress^107^. In addition, milk-EVs modulate the intestinal stem cell activity by increasing the expression of leucine-rich repeat containing G-protein-coupled receptor 5 (Lgr5), a marker for intestinal stem cells^104^. All these activities are important in maintaining homeostasis of intestinal epithelium which is constant renew, tightly regulated by the balance between intestinal stem cell proliferation, migration, and differentiation^108^. In addition, milk-EVs also have been reported to maintain the intestinal barrier integrity^88^. Maintenance of the barrier integrity is important to prevent the entry of microbes and microbial toxin as well as other harmful substances with may lead to intestinal inflammation and other health issues. The expression of secretory immunoglobulin A (SIgA) that plays an important role in maintaining the homeostasis of GI tract by protecting the intestinal epithelium from pathogens and toxins is found to increase upon treatment with milk-EVs^17^. The higher expression of SIgA by porcine jejunal epithelial cells (IPEC-J2) was induced by the Circ-XPO4 cargo in milk-EVs.

### Repair and regeneration

The complex cargo of milk-EVs have been reported in many studies to contribute to the repair and regeneration of intestinal tissue. This is achieved mainly via immune modulation and regulation of the growth, survival, migration, and differentiation of cells involved in intestinal tissue regeneration, including intestinal epithelial cells. Regulation of inflammation is critical in creating a conducive environment for tissue healing. The potential of milk-EVs in suppressing intestinal inflammation has been reported in many studies^109,110^. Lin et al. showed that milk-EVs promoted intestinal epithelial cell proliferation and intestinal epithelial repair in an intestinal organoids models of epithelial injury induced by tumor necrosis factor-α (TNF-α)^111^. In the same study, interrogation using a mice model of dextran sulfate sodium-induced intestinal mucosa damage demonstrated that milk-EVs promote intestinal epithelial regeneration by inducing intestinal epithelial cell proliferation and enhancing the expression of intestinal stem cell markers (Lgr5, olfactomedin 4 (Olfm4) and Achaete-Scute Family BHLH Transcription Factor 2 (Ascl2)).

In addition, milk-EVs were also known to restore the gut barrier integrity^29,88^. In a mice model with malnutrition-induced intestinal barrier dysfunction, the animals that received a daily oral gavage of milk-EVs for 5 consecutive days demonstrated improvement in intestinal barrier function as well as jejunal and ileal villus length^29^. The authors attributed the barrier repair ability of milk-EVs to its ability to modulate certain components of tight junctions, whereby the expression of claudin-3 and occluding were increased but the expression of zonulin-1 maintained. Furthermore, the intestinal epithelial cell proliferation retarded by malnutrition was reverted by milk-EVs which promoted the proliferation of Lgr5+ intestinal stem cells. However, this improvement was only recorded in jejunum, but not the ilium. In another study, milk-EVs improved the colon epithelial tight junction (higher expression of occluding, zonula occluden-1, and junctional adhesion molecule-1) via activation of AMPK and GLP-2/IGF-1 signaling and epithelial mucus barrier through upregulation of mucins in mice with dextran sulfate sodium-induced colitis^88^. The improvement in intestinal permeability prevented the absorption endotoxin which causes liver inflammation. In a LPS-induced necrotizing enterocolitis rat model, milk-EVs reversed the intestinal epithelial cell proliferation and migration inhibition via the activation of ERK/MAPK pathway^68^.

### Immune modulation

Milk-EVs are also well-known for its ability to modulate the immune response. A balanced and regulated GI tract immune system is critical in maintaining the health and proper function of GI tract. These effects can be attributed to various components present in milk-EVs, particularly the immune regulatory molecules that can interact with immune cells and modulate their function. Milk-EVs can interact with immune cells such as macrophages^112^, dendritic cells^113^, and lymphocytes^114,115^. These interactions may lead to alterations in immune cell activation, proliferation, differentiation, as well as cytokine and chemokine production.

Milk-EVs were found to inhibit the activation of naive T cells and their differentiation to memory phenotype and inhibit T helper cell activation without T-cell tolerance or regulatory T-cell induction^114^. However, a separate study found that milk-EVs contain TGF-β which differentiate naive T cells into Th17 cells which provide immunity against infections, but also involve in the pathologies of autoimmune and inflammatory diseases^83^. The study on the effect of milk-EVs on NK cells is still very limited. It has been reported that milk-EVs did not activate the NK cells under normal circumstances, but may activate the NK cells during inflammation^115^.

The effects of milk-EVs on macrophages have been investigated in several studies. Milk-EVs were found to suppress the expression of NF-κB and secretion of pro-inflammatory IL-6 and IL-1β by macrophages^112^. However, contradictory data has been reported whereby a study found that goat milk-EVs polarized the M0 macrophage to pro-inflammatory M1-like phenotype, but it does not affect the cytokine secretion of M1 macrophages^116^. Milk-EVs-induced polarization to M1 phenotype also has been reported in mouse macrophages stimulated with organic dust extract^117^.

The immune modulatory effects of milk-EVs also have been demonstrated in models of GI tract inflammation. In an in vitro model of intestinal inflammation, milk-EVs were found to modulate the production of pro-inflammatory cytokines and chemokines such as IL-1β, IL-8, IL-10, IL-12B, IL-12, IL-17, and TNF-α by the THP-1 cells and colon epithelial cells (Caco-2) induced with IFN-γ and LPS, thus help to mitigate inflammation and maintain immune homeostasis^109^. In porcine jejunal epithelial cells (IPEC-J2) induced with LPS, treatment with milk-EVs reduced the level of IL-18, but increased the levels of IL-1α, IL-6, and IL-8^118^. In another study with mice model of dextran sodium sulfate (DSS)-induced colitis, treatment with milk-EVs led to a reduction in the levels of pro-inflammatory G-CSF, IL-1β, IL-3, IL-6, IL-10, IL-12-p40, IL-12-p70, IL-15, IL-17, MCP-1, MIG, and TNFα as well as higher levels of GM-CSF and anti-inflammatory IL-4, IL-5 and M-CSF^110^, to alleviate the inflammation. In a genetic mouse model of ulcerative colitis, oral intake of milk-EVs ameliorate the disease by suppressing the inflammation^119^.

Necrotizing enterocolitis (NEC) is a common and severe intestinal disease, particularly affecting premature and fragile infants, often resulting in high mortality rates. Bovine milk-EVs have shown promise in preventing intestinal injury by enhancing goblet cell function and endoplasmic reticulum (ER) function. In an experimental mouse model, milk-EVs have been found to prevent NEC by improving mucin expression by goblet cells. Depletion of mucin production from goblet cells occurs early in inflamed intestines before epithelial cell damage. In addition, the study also shown that milk-derived extracellular vesicles decrease myeloperoxidase (MPO) expression in experimental NEC, suggesting a beneficial anti-inflammatory effect associated with the restoration of mucin production^120^.

### Microbiota modulation

The intricate interplay between host physiology and gut microbiota has garnered significant attention in recent research. A novel player in this complex relationship is milk-EVs, which have emerged as potential modulators of the gut microbiota. A study has demonstrated that oral administration of milk-EVs to C57BL/6 mice for 8 weeks modified the gut microbiota composition and modulation of metabolites like short-chain fatty acids (SCFAs). In addition, milk-EVs boosted the expression of key genes involved in intestinal barrier function (Muc2, RegIIIγ) and immune regulation (Myd88, GATA4) within the intestine, alongside increased levels of IgA and sIgA crucial for mucus layer integrity. These findings suggest that milk-EVs play a role in modulating gut microbiota and enhancing intestinal immunity, underscoring their therapeutic potential in gastrointestinal health^98^. Meanwhile, in a study on C57BL/6 mice, milk-EVs were found to increase beneficial gut microbes while reducing harmful bacteria. Serum analysis showed alterations in lipid and amino acid metabolism, along with increased anti-inflammatory factors. KEGG analysis highlighted pathways related to immune function and nutrient metabolism. Overall, milk-EVs positively influenced serum nutrient metabolism without promoting harmful bacteria, suggesting their safety and potential protective effects against diseases^99^. Another investigation has mentioned the effects of two subsets of EVs from commercial cow’s milk (P35K and P100K) on a mouse model of colitis induced by DSS, the analysis shows improved outcomes associated with colitis by modulating gut microbiota, restoring intestinal impermeability, and reducing inflammation. P35K EVs enhanced innate immunity, while P100K EVs downregulated colitis-associated miRNAs, particularly miR-125b, and increased expression of TNFAIP3 (A20). These findings suggest that different milk-EV subsets may offer complementary mechanisms to improve colitis outcomes, with implications for therapeutic strategies and public health^110^. In another study, oral administration of milk-EVs showed protective effects against DSS-induced colitis in mice by modulating gut microbiota composition. Notably, EV treatment increased beneficial bacteria such as Bifidobacterium, Dubosiella, and Lachnoclostridium, along with promoting the production of acetate and butyrate, known for their anti-inflammatory properties. Furthermore, EVs regulated the expression of various genes implicated in inflammation, with a shift towards up-regulating anti-inflammatory genes and down-regulating pro-inflammatory ones. These findings highlight the therapeutic potential of milk-derived EVs as a dietary supplement for managing intestinal inflammation and dysbiosis^121^. A study investigated the therapeutic potential of milk-EVs in IBD, specifically ulcerative colitis (UC), using a mouse model found that milk-EVs contain immune-active proteins and miRNAs involved in immune and inflammatory regulation. Oral administration of milk-EVs ameliorated UC symptoms, including colon shortening, epithelial disruption, inflammatory cell infiltration, and tissue fibrosis. Mechanistically, milk-EVs inhibited the TLR4-NF-κB signaling pathway and NLRP3 inflammasome activation, restored cytokine production balance, and modulated Treg/Th17 cell equilibrium in the inflamed colon. In addition, milk-EVs partially restored the disturbed gut microbiota in UC. Overall, these findings suggest that milk-EVs alleviate colitis by regulating intestinal immune homeostasis and reshaping the gut microbiota^122^.

## Milk-EVs to ameliorate colorectal cancer

**Fig. 2:**
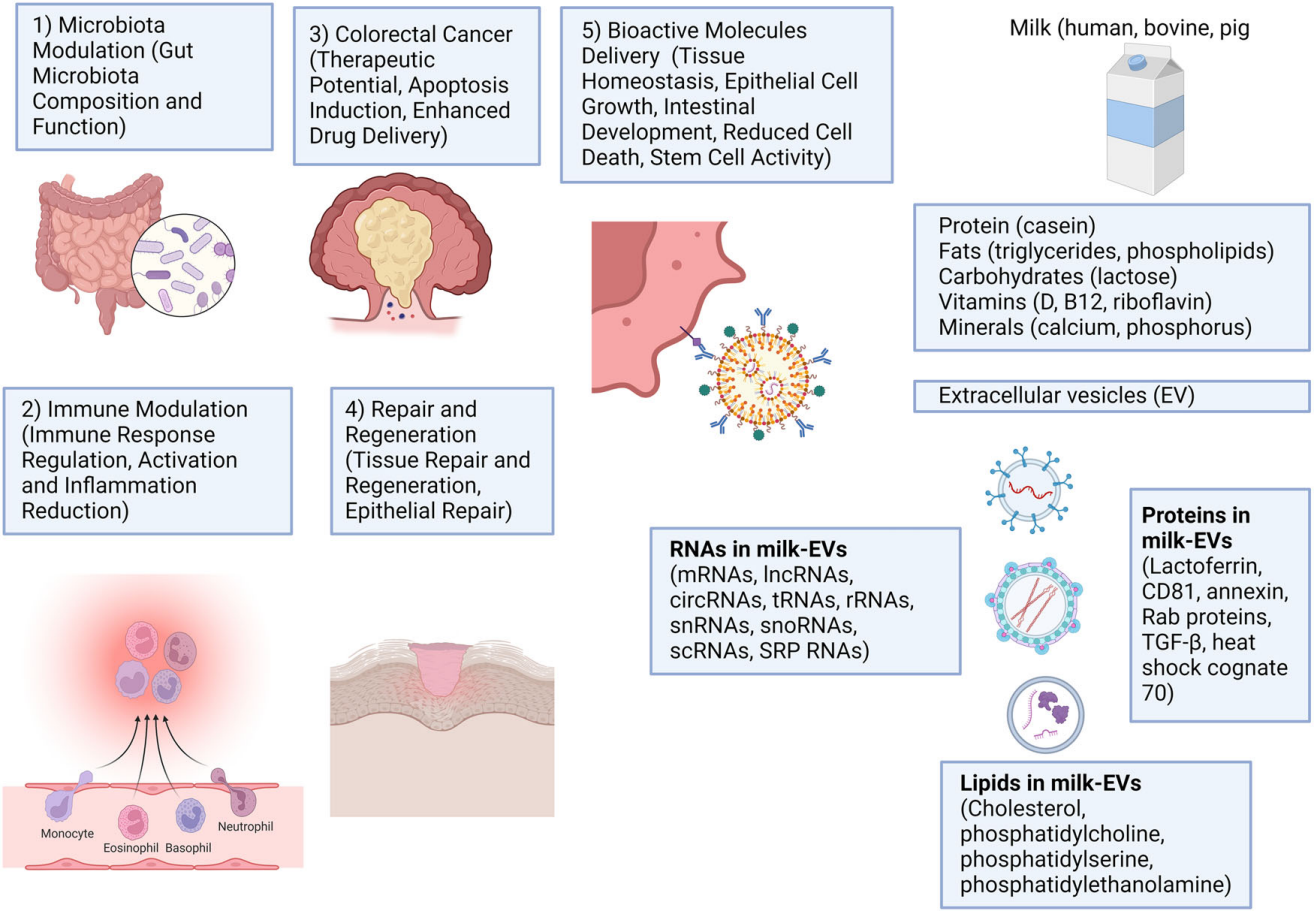
Milk-EVs and their benefits for GI health. Milk-EVs offer numerous benefits for GI health by transporting bioactive molecules like proteins, lipids, and nucleic acids. They support tissue balance, growth of epithelial cells, and gut development while reducing cell death and maintaining barrier strength. Additionally, EVs aid tissue healing by regulating immune responses, cell proliferation, and epithelial repair, as well as altering gut microbiota composition to support beneficial bacteria growth and enhance mucus barrier function. In colorectal cancer, EVs show therapeutic potential through inducing cancer cell apoptosis and enhancing drug delivery with anti-cancer agents, highlighting their potential for creating targeted GI treatments. This figure was created using BioRender.com.

Contradictory results have been reported regarding the effects of milk-EVs on colorectal cancer. In a study, bovine milk-EVs were found to reach the tumor to exert both anti-tumor and pro-tumor effects whereby it shrunk primary tumor by inducing cellular senescence but accelerated the metastasis via activation of epithelial-to-mesenchymal transition^123^. However, in a different study, the authors reported the benefits of buffalo milk- and bovine-EVs to stimulate the death of colon cancer cells (LIM1215)^124^. The anti-tumor effects of milk-EVs is partially attributed by its miR-27b cargo whereby buffalo milk-EVs that are rich in miR-27b were found to promote the apoptosis of colon cancer cells (HCT116 and HT-29) by inducing mitochondrial stress (accumulation of mitochondrial reactive oxygen species), lysosome accumulation, as well as excessive endoplasmic reticulum stress via modulation of PERK/IRE1/XBP1 and CHOP protein pathway^125^. In another study, the anti-tumor effects of bovine milk-EVs was enhanced with the loading of anthocyanidin derived from bilberry^126^. The anthocyanidins-loaded milk-EVs were found to be more potent in inducing the apoptosis of colon cancer cells (HCT116) compared to the naive milk-EVs. Modification of milk-EVs’ cargo or using it as drug carriers to treat colorectal cancer also has been reported in several other studies. Milk-EVs have been utilized to deliver oxaliplatin and indocyanine green to halt the tumor progression^127,128^. Table 5 displays a structured summary of the benefits of milk-EVs to the gastrointestinal system and their potential in various applications. Figure 2 shows the overall health benefits of mEVs for GI Health.

**Table 5:** The benefits and mechanisms of milk-EVs in various application

Category	Benefit	Mechanism	References
Bioactive molecules delivery	Supports tissue homeostasis	Milk-EVs deliver bioactive molecules such as proteins, lipids, and nucleic acids, promoting various cellular functions and signaling pathways within the GI system.	^51,104–108^
	Promotes epithelial cell growth	Milk-EVs have been found to promote the growth of intestinal epithelial cells.	^104–106^
	Supports intestinal tract development	Milk-EVs increase villus height and crypt depth in the intestinal tract.	^105^
	Reduces epithelial cell death	Protect intestinal epithelial cells against oxidative stress.	^107^
	Modulates intestinal stem cell activity	Increases the expression of Lgr5, a marker for intestinal stem cells, aiding in homeostasis.	^104^
	Maintains intestinal barrier integrity	Enhances the expression of secretory immunoglobulin A (SIgA), protecting the intestinal epithelium from pathogens and toxins.	^17,88^
Repair and regeneration	Enhances tissue repair and regeneration	Milk-EVs modulate immune response, regulate growth, survival, migration, and differentiation of intestinal cells, and suppress inflammation.	^109–111^
	Promotes epithelial repair	Induces intestinal epithelial cell proliferation and enhances stem cell marker expression (Lgr5, Olfm4, Ascl2).	^111^
	Restores gut barrier integrity	Increases expression of tight junction proteins (claudin-3, occludin, ZO-1) and promotes epithelial cell proliferation in malnutrition-induced dysfunction models.	^29,88^
Immune modulation	Regulates immune response	Milk-EVs interact with macrophages, dendritic cells, and lymphocytes, altering activation, proliferation, differentiation, and cytokine production.	^112–115^
	Inhibits T-cell activation	Inhibits naive T-cell differentiation to memory phenotype and T helper cell activation.	^114^
	Modulates macrophage polarization	Polarizes M0 macrophages to pro-inflammatory M1-like phenotype.	^116,117^
	Reduces inflammation	Decreases pro-inflammatory cytokine levels and increases anti-inflammatory cytokines in GI tract models.	^109,110,119^
Microbiota modulation	Alters gut microbiota composition and function	Modulates gut microbiota, increases beneficial bacteria, reduces harmful bacteria, and enhances intestinal immunity.	^98,99,110,121,122^
	Enhances mucus barrier	Increases mucin expression by goblet cells, improving mucus layer integrity and protecting against pathogens.	^120^
Cancer	Therapeutic potential in colorectal cancer treatment	Contradictory effects; milk-EVs can induce cellular senescence in primary tumors and apoptosis in colon cancer cells, and can be used as drug carriers for enhanced anti-tumor effects.	^123–128^
	Induces apoptosis in cancer cells	Promotes apoptosis via mitochondrial stress and endoplasmic reticulum stress modulation.	^125^
	Enhances drug delivery	Milk-EVs can be loaded with anti-cancer agents (e.g., anthocyanidins, oxaliplatin) for targeted delivery to tumors.	^126–128^

## Perspectives and conclusion

The study of milk-EVs presents exciting prospects for advancing GI health and therapeutics. As our understanding of the composition, function, and absorption mechanisms of milk-EVs deepens, their potential applications in clinical settings are likely to expand. Future research endeavors could focus on elucidating the specific roles of individual components within milk-EVs, such as proteins, lipids, and nucleic acids, in mediating their therapeutic effects. Moreover, exploring the interplay between milk-EVs and the gut microbiota holds promise for novel therapeutic strategies aimed at modulating microbial composition and activity to promote GI health. By elucidating the mechanisms by which milk-EVs interact with gut microbes and influence their functions, researchers may uncover new avenues for the treatment of dysbiosis-related conditions and enhancement of gut barrier integrity. In addition, leveraging milk-EVs as delivery vehicles for bioactive molecules and therapeutic agents could revolutionize oral drug delivery, offering targeted and efficient treatment options for GI diseases while minimizing systemic side effects. Furthermore, as technological advancements continue to improve our ability to isolate, characterize, and manipulate milk-EVs, novel approaches for enhancing their therapeutic efficacy and bioavailability may emerge. Strategies such as engineering milk-EVs to enhance targeting specificity, prolong circulation time, or increase cargo loading capacity could further enhance their utility in clinical applications. In addition, ongoing efforts to optimize EV isolation and purification methods, as well as standardize characterization protocols, will be crucial for ensuring reproducibility and scalability in future clinical translation. The future of milk-derived EVs in GI health and therapeutics appears promising, with opportunities for innovation and discovery across multiple fronts. However, realizing this potential will require continued interdisciplinary collaboration, robust preclinical and clinical studies, and a concerted effort to overcome existing challenges in EV research and translation. Ultimately, the ongoing exploration of milk-EVs represents a dynamic and rapidly evolving field with significant implications for the future of GI healthcare.

In conclusion, milk-EVs present a promising avenue for promoting GI health and addressing associated disorders. These EVs are rich in bioactive molecules, including proteins, lipids, and nucleic acids, which contribute to their diverse functional roles within the GI system. Their ability to resist degradation in the harsh conditions of the stomach and traverse the intestinal epithelium for absorption highlights their potential as effective delivery vehicles for bioactive compounds. Once absorbed, milk-EVs have been shown to modulate immune responses, promote tissue repair and regeneration, and modulate gut microbiota composition, thereby contributing to overall GI health. While research in this field is ongoing, milk-EVs hold considerable therapeutic potential for conditions such as inflammatory bowel disease and colorectal cancer. However, further investigation is warranted to elucidate their mechanisms of action, optimize delivery methods, and evaluate their safety and efficacy in clinical settings, paving the way for their translation into targeted therapeutic interventions for GI disorders.

## Abbreviation

CD: cluster of differentiation
CHOP: C/EBP homologous protein
circRNA: circular RNA
DNA: deoxyribonucleic acid
DSS: dextran sulfate sodium
EMT: epithelial-to-mesenchymal transition
ERK1/2: extracellular signal-regulated kinase 1/2
EV: extracellular vesicle
FcRn: Fc receptor
GI: gastrointestinal
GM-CSF: granulocyte-macrophage colony-stimulating factor
HPLC: high-performance liquid chromatography
IBD: inflammatory bowel disease
IL: interleukin
IMF: infant milk formulation
LC-MS: liquid chromatography-mass spectrometry
lncRNA: long noncoding RNA
M-CSF: macrophage colony-stimulating factor
MCP-1: monocyte chemoattractant protein-1
MIG: monokine induced by gamma interferon
miRNA: microRNA
mRNA: messenger RNA
MS: mass spectrometry
NGS: next-generation sequencing
NLRP3: NOD-like receptor family pyrin domain containing 3
p38 MAPK: p38 mitogen-activated protein kinase
PEG: polyethylene glycol
Rab: Ras-associated binding
RNAs: ribonucleic acids
rRNA: ribosomal RNA
SEC: size-exclusion chromatography
scRNA: small cytoplasmic RNA
snRNA: small nuclear RNA
snoRNA: small nucleolar RNA
SRP RNA: signal recognition particle RNA
Th17: T helper 17
TGF-β: transforming growth factor beta
TNFAIP3: tumor necrosis factor alpha-induced protein 3
TLR4-NF-κB: Toll-like receptor 4-nuclear factor kappa B
tRNA: transfer RNA
Treg: regulatory T cell
UC: ultracentrifugation
